# GARP Correlates With Tumor-Infiltrating T-Cells and Predicts the Outcome of Gastric Cancer

**DOI:** 10.3389/fimmu.2021.660397

**Published:** 2021-08-06

**Authors:** Sutian Jiang, Yifan Zhang, Xiaojing Zhang, Bing Lu, Pingping Sun, Qianqian Wu, Xuzhong Ding, Jianfei Huang

**Affiliations:** ^1^Department of Clinical Biobank, Affiliated Hospital of Nantong University, Nantong, China; ^2^Department of Pathology and Pathophysiology, School of Medicine, Nantong University, Nantong, China; ^3^Clinical Medicine, Xian Medical University, Xi’an, China; ^4^Translational Medicine Center, The Affiliated Kezhou People’s Hospital of Nanjing Medical University, Kezhou, China

**Keywords:** GARP, mIHC, TMA, TME, gastric cancer

## Abstract

Accepting the crucial role of the immune microenvironment (TME) in tumor progression enables us to identify immunotherapeutic targets and develop new therapies. Glycoprotein A repetitions predominant (GARP) plays a vital part in maintaining regulatory T cell (Treg)-mediated immune tolerance. The impact of GARP in TME of gastric cancer is still worth exploring. We investigated public genomic datasets from The Cancer Genome Atlas and Gene Expression Omnibus to analyze the possible role of GARP and its relationship with TME of gastric cancer. Fluorescence-based multiplex immunohistochemistry and immunohistochemistry for T-cell immune signatures in a series of tissue microarrays were used to validate the value of GARP in the TME. We initially found that GARP expression was upregulated in gastric carcinoma cells, and diverse levels o3f immune cell infiltration and immune checkpoint expression were detected. Gene expression profiling revealed that GARP expression was related to the TME of gastric cancer. GARP upregulation was usually accompanied by increased FOXP3+ Treg and CD4+ T cell infiltration. In addition, GARP expression had positive relationships with CTLA-4 and PD-L1 expression in gastric cancer. Cox regression analysis and a nomogram highlighted that the probability of poor overall survival was predicted well by GARP or GARP+CD4+ T cell. Taken together, this research underlines the potential effect of GARP in regulating survival and tumor-infiltrating T-cells. In addition, the function of CD4+ T cell immune signatures in the prognosis can be clinically meaningful, thereby providing a new idea for the immunotherapeutic approach.

## Introduction

Gastric cancer (GC) patients which received the conventional treatment at the same stage usually showed heterogeneous clinical prognosis (1, 2). Therefore, we need a prognostic signature that is different from the previous staging system to accurately predict the outcome of patients and better guide adjuvant therapy (2–4). Tumor-associated immune cells in the tumor microenvironment have been demonstrated to play a vital part in tumor development and affect the clinical outcomes of patients (5, 6). Although remarkable progress has been made in cancer treatment through the blockade of CTLA-4 or PD-1 signaling using monoclonal antibodies (mAbs), most patients do not respond to immunotherapy because of primary or acquired drug resistance (7, 8). Therefore, a better understanding of the markers associated with T cells in the TME is meaningful for deciphering the mechanisms of immunotherapy and identifying new therapeutic targets (9, 10).

The transforming growth factor-β (TGF-β) superfamily is an important family of regulatory cytokines with multiple functions in development, immunity, and cancer (11). GARP (commonly known as leucine-rich repeat-containing 32) is a cell surface docking receptor for latent TGF-β, and also has been studied as a non-signal receptor on the surface of Tregs, platelets, and certain cancer cells (12–14). GARP forms a complex with integrin and releases active TGF-β from the cell surface, thereby enhancing the inhibitory phenotype of Tregs (15–17). It has been reported that GARP is overexpressed in colon, lung, and breast cancers, and patients with high GARP expression tend to have a poor prognosis (12, 18). Therefore, the roles of GARP in the immune microenvironment of gastric cancer and prognosis are worthy of further exploration. In the present study, we combined experiment and bioinformatic technique to further characterize the potential impact of GARP in regulating survival and the TME of gastric cancer, thereby finding TME-associated prognostic signature.

## Methods

### Bioinformatic Analysis

#### Evidence From the Public Database

TCGA clinical and RNA-Seq data for GC patients, including 375 tumor samples, 27 paracancerous samples, and 32 normal samples, were download from Genome Data Commons (https://portal.gdc.cancer.Gov/). We excluded data missing key information, such as overall survival (seven cases), age (four cases), and lymph node metastasis (two cases). Our research meets the publishing requirements provided by TCGA. We also obtained an additional GEO dataset, GSE84437, which contained 434 GC patients with survival information (https://www.ncbi.nlm.nih.gov/geo/).

#### TISIDB Analysis

TISIDB is a website for comprehensive research on the immune microenvironment that integrates tumor immunology with multiple types of data resources (http://cis.hku.hk/TISIDB/) (19). In TISIDB, we can use literature mining and high-throughput data analysis to clarify the roles of genes of interest in tumor-immune interactions. We analyzed the effect of GARP expression on the prognosis of patients with gastric cancer and its connections with the clinicopathological parameters and immune subtypes of gastric cancer.

#### TIMER Database Analysis

TIMER (v.2.0.) used a deconvolution statistical method to infer the prevalence of tumor-infiltrating immune cells (TIICs) based on the gene expression profile (https://cistrome.shinyapps.io/timer/), The database used TCGA data from 10897 samples of 32 cancers to approximate the abundance of TIICs. We performed a gene module to assess the association between GARP expression in gastric cancer and TIICs, including B cells, CD4 + T cells, CD8 + T cells, neutrophils, macrophages, and dendritic cells.

#### CIBERSORT Estimation

CIBERSORT is an analytical tool for deconvolution of the expression matrix of immune cell subtypes based on the principle of linear support vector regression (https://cibersort.stanford.edu/index.php) (20). We used the CIBERSORT database to explore the infiltration levels of 22 immune cells in gastric cancer. Standard annotation files were utilized to generate gene expression datasets. CIBERSORT approximates the p-value *via* Monte Carlo sampling and deconvolution to determine the credibility of the results. We grouped the data downloaded from the TCGA database according to the immune subtypes obtained *via* single-sample Gene Set Enrichment Analysis (ssGSEA) to evaluate the infiltration of immune cells in different immune subtypes.

#### Gene Set Enrichment Analysis (GSEA) and Unsupervised Clustering

GSEA is a tool for analyzing genome-wide expression profiling data (21). The basic idea is to use a predefined set of genes, sort the genes according to the degree of differential expression between two sample types, and then test whether the predefined set of genes is enriched at the top or bottom of the sorted table. The samples were first grouped according to phenotypes, and then the differential gene sets were selected according to the group. GSEA determined which group the gene sets assembly chose. In this case, the gene sets were associated with the phenotypic grouping. We downloaded RNA-Seq data for gastric cancer from the TCGA database. Then, we performed GSEA using R (v.3.5.3) to identify signaling pathways that were differentially activated in gastric cancer. The threshold was determined using the following parameters: false-discovery rate (FDR) < 0.05 and P < 0.05.

The infiltration levels of the different immune cell populations were determined *via* single-sample GSEA (ssGSEA) using the R Bioconductor package Gene Set Variation Analysis with the default parameters. The ssGSEA algorithm is a rank-based method that defines a score representing the degree of absolute enrichment of a particular gene set in each sample. GSEA was performed on each sample using transcriptome data and clinical data downloaded from the TCGA database. We obtained the immune cells, immune-related gene sets, and immune-related pathways of each sample, thereby permitting the immune activity of each sample to be evaluated using the CIBERSORT and ESTIMATE algorithms. Unsupervised clustering classifies the samples into distinct subtypes according to the immune cell infiltration pattern of each sample. The unsupervised clustering “Pam” method in accordance with Euclidean and Ward’s linkage was used in our analysis, executed by using the “ConsensuClusterPlus” R package, and repeated 1,000 times to ensure the classification stability.

#### Protein-Protein Interactions

STRING is a database of known and predicted protein-protein interactions (https://string-db.org/) (22). The interactions include direct (physical) and indirect (functional) associations. They stem from computational prediction, knowledge transfer between organisms, and interactions aggregated from other (primary) databases. We used the STRING database to build a protein network of interactions between GARP and related immune signatures.

### Human Tissue Samples and Patient Clinical Information

The tissue microarray (TMA) (176 gastric cancer tissues, 52 normal gastric mucosa tissues) used in this research was prepared by the Department of Clinical Biobank of the Affiliated Hospital of Nantong University. A core on the TMA represents a sample with a diameter of 2 millimeters. We averaged the results of multiple samples from the same patient. This research retrospectively analyzed the clinicopathological features and prognoses of the patients. We collected clinicopathological information from the patients’ medical records. The patients had not received radiotherapy, chemotherapy, or biological immunotherapy before surgery. This research protocol was approved by the Human Research Ethics Committee of the Affiliated Hospital of Nantong University (Jiangsu, China).

### Immunohistochemistry (IHC)

Formalin-fixed, paraffin-embedded TMA sections were deparaffinized and rehydrated using alcohol and xylene. TMA sections were heated using a microwave in sodium citrate buffer (0.01 M, pH 6.0) to repair antigen. The sections were incubated with 5%BSA to quench endogenous peroxidase activity and then with rabbit anti-PD-L1 (13684S, Cell Signaling Technology) and mouse anti-CTLA-4 antibodies (NB10064849, NOVUS). An EliVision Plus DAB Kit (Kit‐0015; Maxim Biotechnologies, Fuzhou, China) was used to analyze the result of antibody binding. The results of TMA staining were assessed using the semiquantitative H‐score method by a pathologist who was blinded to the clinical information of the patients. The staining intensity score was multiplied by the percentage of positively stained cells to calculate the total score, which ranged from 0 to 300.

### Fluorescence-Based Multiplex Immunohistochemistry (mIHC)

TMA sections were heated using a microwave in AR6 buffer (AR600, AKOYA) to repair antigen. MIHC staining was performed after the secondary antibody was added, and then the antigen was repaired *via* heat induction and cooling. The nucleus was stained with DAPI and sealed. The slides were scanned using the Vectra 3.0 automated quantitative pathology imaging system to detect and measure the positive rate of biomarkers. The cores containing both tumor and stroma were captured with a ×20 Olympus lens objective. Using inForm^®^ Cell Analysis software, we train machine-learning algorithms to segment the images into tissue areas of cancerous cells and stromal cells, to segment individual cells by DAPI counterstaining, and to accurately identify and quantify the phenotypes of those cells in all high-power fields within the entire tissue section.

The following primary antibodies were used in this study: rabbit anti-GARP (orb36818, BIORBYT), rabbit anti-CD3 (85061S, Cell Signaling Technology), rabbit anti-CD4 (ab133616, Abcam), rabbit anti-CD8 (ab83278, Abcam), and mouse anti-FOXP3 (ab20034, Abcam). The secondary antibody was Opal™ polymer HRP Ms+Rb (ARH1001EA, Perkin Elmer). Fluoroshield with DAPI (F6057, Sigma) was used to stain nuclei and seal the slices.

### Statistical Analysis

Student’s 𝑡-test was used to compare GARP protein expression between tumor and non-tumor tissue samples. Pearson’s χ^2^ test was performed to determine the correlation between GARP expression and clinicopathologic parameters. Cox regression models were used to identify prognostic factors. We used the “rms” R package to formulate nomograms, which can predict the probability of 1-year, 3-year, and 5-year overall survival for GC patients. R software (v.3.6.0), SPSS (v.17.0), GraphPad Prism (v.5.0), and Strawberry Perl (v.5.30.1) were used in the early data processing of this study. For all tests, P < 0.05 was considered statistically significant.

## Results

### Immune Microenvironment Grouping of Patients With Gastric Cancer

**Figure 1:**
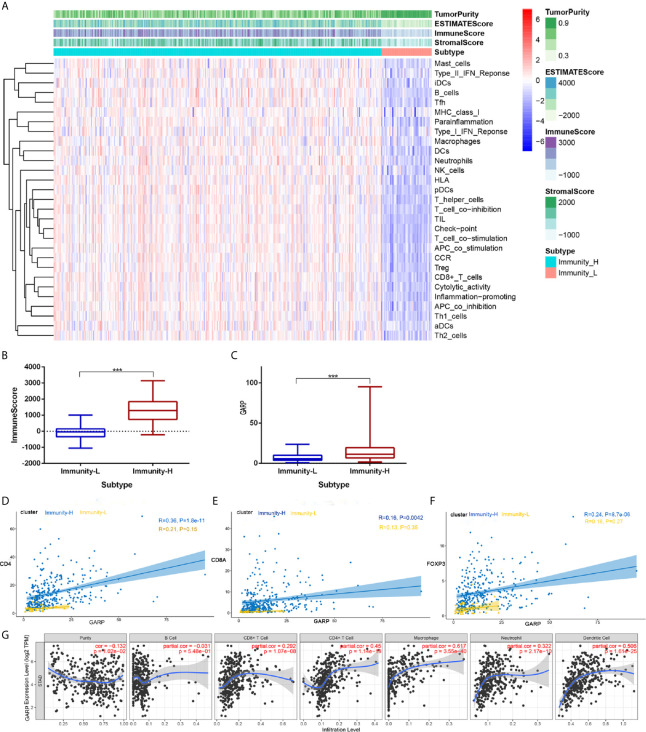
**(A)** Immune cell score, stroma cell score, comprehensive scores of immune and stromal cells, and tumor purity score in different immunity groups. **(B)** Analysis of the difference of the immune score between the high and low immunity groups. **(C)** Analysis of the difference of glycoprotein A repetitions predominant (GARP) expression between the high and low immunity groups. **(D–F)** The relationships of GARP with CD4, CD8A, and forkhead box protein 3 (FOXP3) in different immune groups. Immunity-L, low immunity group; Immunity-H, high immunity group. ***, P < 0.001. **(G)** Correlations between GARP expression and immune cells infiltration levels in TIMER.

TME is mainly composed of tumor-infiltrating immune cells, extracellular matrix, and secreted factors that are highly related to overall survival and treatment response (23). We used the CIBERSORT and ESTIMATE algorithms to evaluate each TCGA sample by scoring immune cells, stromal cells, and tumor purity. Besides, we divided TCGA samples into high and low immunity groups *via* the unsupervised clustering “Pam” method. Compared with that in the high immunity group, the immune score was significantly lower in the low immunity group, whereas the tumor score was higher in the low immunity group (P < 0.05) (**Figures 1A** **, B**). In addition, GARP expression was significantly increased in the high immunity group (P < 0.001) (**Figure 1C**). Then, we investigated the link between GARP expression and the markers of CD4+ T cells, CD8+ T cells, and Tregs in different immune groups. We found that GARP expression was related to CD4, CD8A, and FOXP3 expression, and the correlation was stronger in the high immunity group than in the low immunity group (P < 0.05) (**Figures 1D–F**). To determine whether GARP was involved in the activation of Treg in gastric cancer, we subdivided the GARP high/low group into a TGF-β1 low and high group, and then made a Kaplan-Meier curve with overall survival (**Supplementary Figure 1**). We also divided the GARP high/low group into a FOXP3 low and high group. However, the P-value of Kaplan-Meier curve with overall survival was no statistical significance. In TIMER database, there was a significant link between GARP expression and the levels of CD4+ T cells (Pearson correlation = 0.450, P < 0.05), CD8+ T cells (Pearson correlation = 0.290, P < 0.05), macrophages (Pearson correlation = 0.617, P < 0.05), neutrophils (Pearson correlation = 0.322, P < 0.05), and dendritic cells (Pearson correlation = 0.506, P < 0.05) (**Figure 1G**). These findings illustrated the relationship of GARP with the immune microenvironment in gastric cancer.

### High Expression of GARP Is Associated With Clinicopathological Features

**Figure 2:**
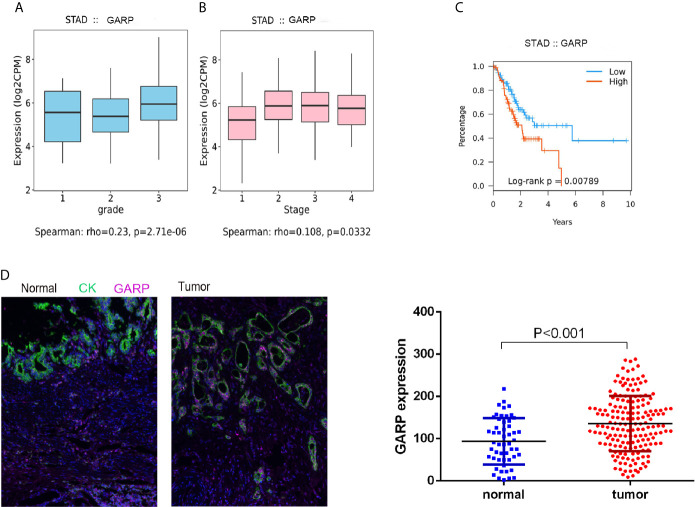
**(A, B)** Increased GARP expression was significantly associated with unfavorable histologic grade and advanced clinical stage. **(C)** The Kaplan–Meier curve for overall survival in patients with gastric cancer in the TISIDB database. The cutoff point was based on the median. **(D)** Fluorescence-based multiplex immunohistochemistry revealed differences in glycoprotein A repetitions predominant (GARP) expression between tumor and normal samples. Cytokeratin (CK) was used to identify epithelial cells in tumor samples and to define tumor and stroma. All images were obtained using 20 × zoom and were scaled digitally.

Although histological classification or clinical staging can well help to predict the prognosis of GC patients, other markers are needed to detect tumor progression. GARP expression was correlated with the tumor grade (P < 0.05) and stage (P < 0.05) (**Figures 2A** **, B**). In the TISIDB database, the samples were classified into distinct subtypes according to the median GARP mRNA levels. High GARP expression in patients with gastric cancer was associated with a worse prognosis (P < 0.05) (**Figure 2C**). A great amount of transcriptomic data may not be translated into proteins. We next performed fluorescence-based mIHC using TMA and determined that GARP protein levels significantly differed between tumor and normal tissues (P < 0.05) (**Figure 2D**). MIHC staining was combined with multispectral image analysis to estimate the positive rate of GARP in a cohort of GC patients. Cytokeratin (CK) was used to identify epithelial cells in tumor samples and to define tumor and stroma, and DAPI was used to stain nuclei. Machine-learning algorithms were trained to distinguish between different tissues (tumor tissue, stroma, and no tissue) and cell phenotypes (tumor cell, and immune cell).

Then, we analyzed whether GARP expression levels were associated with clinicopathological features, including gender, age, tumor size (T), lymph node metastasis (N), distant metastasis (M), TNM stage, tumor differentiation, preoperative serum carcinoembryonic antigen (CEA) levels, and preoperative serum carbohydrate antigen 19-9 (CA19-9) levels. 176 GC patients were divided into the GARP-high group (88 cases) and GARP-low group (88 cases) based on the median GARP expression. From our analysis, we observed marked correlations of GARP expression with tumor size (P < 0.05), distant metastasis (P < 0.05), and TNM stage (P < 0.001) (**Table 1**). We performed Bonferroni adjustment for multiple comparisons of clinicopathologic characteristics in **Supplementary Table 1**.

**Table 1:** Association of glycoprotein a repetitions predominant (GARP) expression levels with clinicopathological characteristics in patients with gastric cancer.

Characteristic	Total No.	Low or No Expression, No. (%)	High Expression, No. (%)	Pearson χ2	P-Value
Total No.	176	88 (50.00)	88 (50.00)		
Sex				1.031	0.398
Man	128	61 (47.66)	67 (52.34)		
Female	48	27 (56.25)	21 (43.75)		
Age (year)				0.211	0.760
≤60	73	38 (52.05)	35 (47.95)		
>60	103	50 (48.54)	53 (51.46)		
Differentiation				1.729	0.421
Well	9	5 (55.55)	4 (44.45)		
Middle	47	26 (55.32)	21 (44.68)		
Poor	97	43 (44.33)	54 (55.67)		
Unknown	23	14	9		
T				15.532	0.001*
Tis+T1	26	19 (73.08)	7 (26.92)		
T2	41	27 (65.85)	14 (34.15)		
T3	100	38 (38.00)	62 (62.00)		
T4	9	4 (44.44)	5 (55.56)		
N				5.311	0.150
N0	77	45 (58.44)	32 (41.56)		
N1	25	13 (52.00)	12 (48.00)		
N2	38	14 (36.84)	24 (63.16)		
N3	36	16 (44.44)	20 (55.56)		
M				5.724	0.032*
M0	164	86 (52.44)	78 (47.56)		
M1	12	2 (16.67)	10 (83.33)		
TNM				18.683	<0.001*
I	48	35 (72.92)	13 (27.08)		
II	56	28 (50.00)	28 (50.00)		
II	60	23 (38.33)	37 (61.67)		
IV	12	2 (16.67)	10 (83.33)		
Preoperative				2.666	0.143
CEA, ng/m1	76	46 (60.53)	30 (39.47)		
≤5	22	9 (40.91)	13 (59.09)		
>5	78	33	45		
Unknown					
Preoperative				1.569	0.332
CA199, u/ml	76	40 (52.63)	36 (47.37)		
≤37	11	8 (72.73)	3 (27.27)		
>37	89	40	49		
Unknown					

*P < 0.05. T, tumor size; N, lymph node metastasis; M, distant metastasis; CEA, carcinoembryonic antigen; CA19-9, carbohydrate antigen 19-9.

### The Relationship Between GARP and TIICs

**Figure 3:**
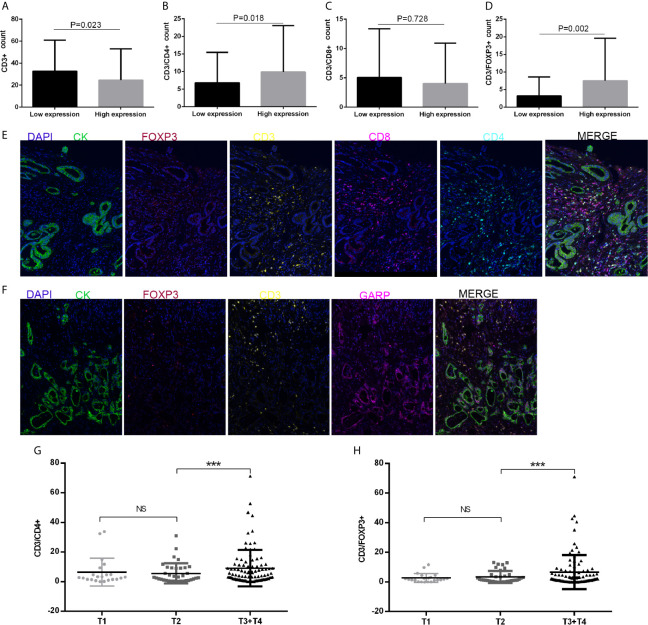
**(A–D)** CD3+ T cell, CD4+ T cell, CD8+ T cell, and FOXP3+ regulatory T cell (Treg) infiltration levels in patients with low or high glycoprotein A repetitions predominant (GARP) expression. **(E, F)** Representative fluorescence-based multiplex immunohistochemistry images. **(E)** A staining panel was developed to visualize CD3, CD4, CD8, FOXP3, and CK simultaneously on the same tissue slide. **(F)** A staining panel was developed to visualize CD3, GARP, FOXP3, and CK simultaneously on the same tissue slide. **(G, H)** Tumor size (T) was associated with changes in CD4+ T cell and FOXP3+ Treg infiltration. Ns, P > 0.05; ***, P < 0.001.

We performed computational imaging techniques to evaluate multiple lymphocyte markers at the same time, allowing spatial analysis of different T cell populations in the same sample tissue section (**Figures 3E** **, F**). TMA sections were developed to visualize CD3, CD4, CD8, FOXP3, GARP, and CK simultaneously on a cohort of gastric cancer samples. These markers were indicated as signatures for T cells (CD3, CD4, CD8, and FOXP3). Our results demonstrated that nearly all samples had varying degrees of immune cell infiltration. Then, we analyzed whether TIIC counts differed between patients with gastric cancer according to GARP expression. Our samples were divided into two groups based on the median GARP expression level. CD3+ T cell infiltration was significantly suppressed in the high GARP expression group compared with that in the low GARP expression group (P < 0.05) (**Figure 3A**). In addition, CD4+ T cell (P < 0.05) and FOXP3+ Treg infiltration (P < 0.05) were obviously enhanced in the high expression group, whereas CD8+ T cell infiltration did not differ significantly between the two groups (P = 0.728) (**Figures 3B** **–D**). In addition, our analysis illustrated that the levels of immune cell infiltration were vastly correlated with tumor size (T) (**Figures 3G** **, H**). According to GARP high/low expression, we subdivided our data to reveal the effect of GARP within T groups on levels of immune cell infiltration. CD4+ T cell and FOXP3+ Treg infiltration were slightly higher in GARP high group (**Supplementary Figure 2**). From our exploration, we can conclude that GARP, as a surface molecule of Tregs, is associated with the infiltration of CD4+ T cell and FOXP3+ Treg but not that of CD8 + T cell.

### GARP Upregulation or GARP+CD4+ T Cell Is an Independent Prognostic Factor for Poor Overall Survival

**Figure 4:**
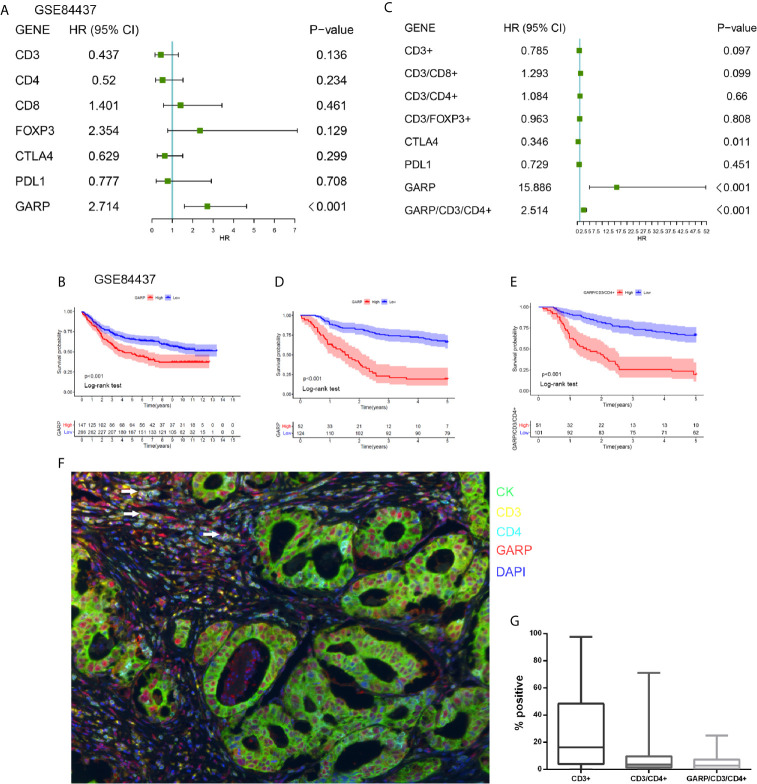
**(A)** A forest plot visualizing the impact of the immune microenvironment (TME)-associated signatures and GARP on overall survival (OS) in the GSE84437, as evaluated using Cox univariate tests. **(B)** Kaplan–Meier curve for the high and low expression groups in the GSE84437. **(C)** A forest plot visualizing the impact of the immune microenvironment (TME)-associated signatures and GARP on overall survival (OS) in our cohort, as evaluated using Cox univariate tests. **(D)** Kaplan–Meier curve for the high and low expression groups in our cohort. **(E)** Kaplan-Meier plot visualizing survival associations of GARP+CD4+ T cell. The optimal cutoff point was obtained from X-tile 3.6.1 software. **(F)** Representative fluorescence-based multiplex immunohistochemistry images of the rate of positivity for GARP+CD4+ T cell in tissue microarray sections. **(G)** A plot shows the rate of positivity for CD3+ T cell, CD4+ T cell, and GARP+ CD4+ T cell. White arrows in the picture that point to the GARP+CD4+ cells.

We utilized a cohort of 434 GC patients (GSE84437) to further comprehend the survival mechanism associated with the relationship between GARP and T-cell immune signatures. Cox regression analysis showed only GARP can be used as an independent factor affecting the prognosis of gastric cancer compared with other immune molecules (P < 0.001) (**Figures 4A** **, B**). In our research cohort, GARP upregulation in gastric cancer was a prognostic factor for poor overall survival (P < 0.001) (**Figures 4C** **, D**). We evaluated GARP expression levels in stroma and tumor cells, respectively. Immunofluorescence results showed the positive staining of GARP in CD4+ T cells (**Figures 4F** **, G**). Additionally, high proportions of GARP+CD4+ T cells from all T cells translated to the inferior outcome (P < 0.05) (**Figure 4E**). On the contrary, CD3+ T cell, CD4+ T cell, CD8+ T cell, and FOXP3+ Treg were not associated with the survival of gastric cancer.

### Construction and Evaluation of a Nomogram for Overall Survival

**Figure 5:**
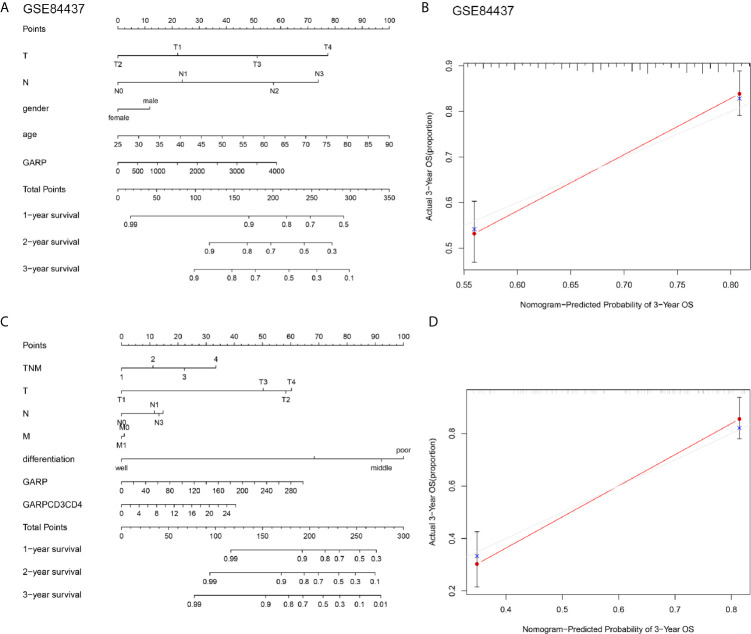
**(A, C)** Nomograms were constructed with the GSE84437 and our research cohort for predicting the probability of 1-year, 3-year, and 5-year overall survival for GC patients. **(B, D)** Calibration plots of the nomograms for predicting the probability of overall survival at 3 years in the GSE84437 and our research cohort. The grey line represents the ideal nomogram, and the red line represents the observed nomogram.

Cox regression analyses were conducted to exhibited that GARP or GARP+CD4+ T cell could serve as an independent predictor of patients’ overall survival after adjusted by TME-associated signatures in multiple GC cohorts (**Figure 4**). Based on logistic regression, we generated a nomogram that integrated GARP, GARP+CD4+ T cell, and other clinicopathological features, including tumor size, lymph node metastasis, distant metastasis, TNM stage, tumor differentiation to predict the probability of 1-year, 3-year, and 5-year overall survival for GC patients with the GSE84437 and the experimental cohort (**Figures 5A** **, C**). The calibration plots revealed the probability of a 3-year survival rate is well predicted in the GSE84437 cohort and the experimental cohort (**Figures 5B** **, D**).

### Exploration of the Molecular Mechanism of GARP

**Figure 6:**
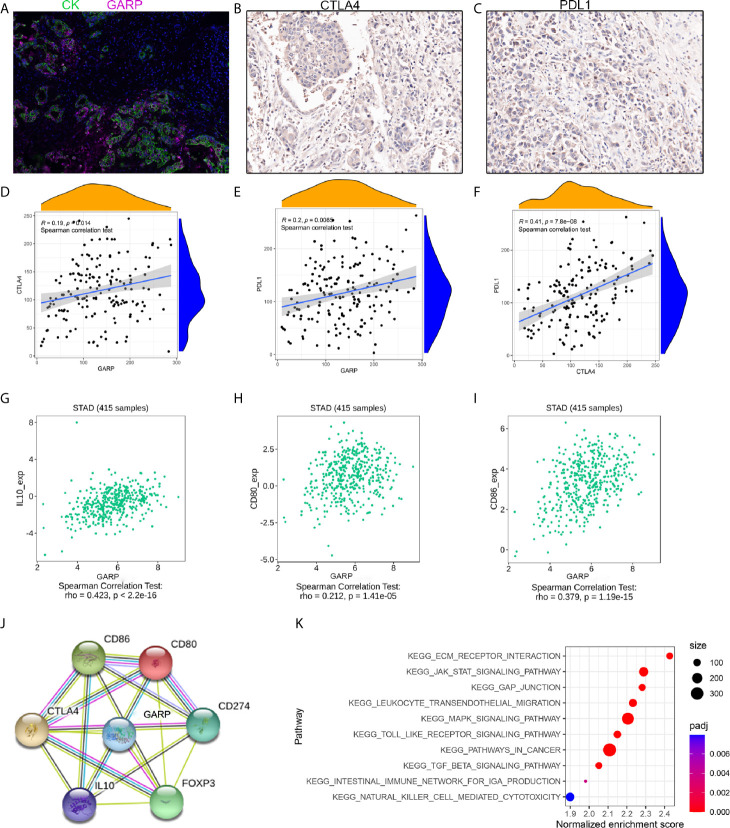
**(A–C)** Representative images of GARP, PD-L1, and CTLA-4 staining in tissue microarray sections of tumor tissues. **(D–F)** The relationships between GARP and PD-L1 expression, GARP and CTLA-4 expression, and PD-L1 and CTLA-4 expression. **(G–I)** GARP expression was correlated with CD80, CD86, and IL-10 expression in the TISIDB database. **(J)** Protein interaction network in the STRING online database. CD274: PD-L1. **(K)** KEGG pathway analysis revealed 10 positively correlated pathways.

We investigated whether the prognostic effect of GARP is related to immune checkpoints in gastric cancer. IHC was performed to explore the expression of PD-L1 and CTLA-4 in gastric cancer and then analyzed their correlations with GARP expression. As presented in **Figures 6A–F**, GARP expression was associated with CTLA-4 (P < 0.05) and PD-L1 expression (P < 0.05). Meanwhile, a positive relationship between CTLA-4 and PD-L1 expression was noted in gastric cancer (P < 0.05).

As the surface receptor of Treg, CTLA-4 can bind CD80/CD86 on the antigen-presenting cells (APC) (24). Then, APC interacts with activated antigen-specific effector T cell, thereby transforming these cells into induced Tregs (25). Induced Treg exerts an immunosuppressive effect by secreting TGF-β and IL-10. In TISIDB, we found that GARP expression was correlated with CD80, CD86, and IL-10 expression (**Figures 6G** **–I**). We confirmed this correlation in the TCGA cohort (**Supplementary Figures 1E, F**). In addition, GARP, CTLA-4, PD-L1, FOXP3, CD80, CD86, and IL-10 formed a protein-protein network (**Figure 6J**).

We divided 176 GC patients into the GARP-high group and GARP-low group based on the median GARP expression. GSEA analysis screened the differential genes according to the sample groups, and then enriched the genes. The results showed that gene sets were enriched with GARP upregulation in GC samples. We selected 10 KEGG pathways with significant differences according to the normalized enrichment score (FDR < 0.25, NOM P < 0.05). Specifically, the following pathways were significantly enriched in the high expression phenotype: ECM-receptor interaction, GAP junction, leukocyte transendothelial migration, the JAK-STAT signaling pathway, the MAPK signaling pathway, pathways in cancer, the TGF-β signaling pathway, the Toll-like receptor signaling pathway, the intestinal immune network for IgA production, and natural killer cell-mediated cytotoxicity (**Figure 6K**).

## Discussion

The genome resources of the public database provide a unique platform for us to further explore the molecular characteristics of different cancers (24). Our research explored the relationship between GARP and the immune microenvironment of gastric cancer based on TCGA and GEO data. The success of cancer immunotherapy has revealed that immune cells, especially T cells, can be helpful in eliminating tumor cells (26). Wang Yu Cai et al. revealed that evaluating the TME components can predict survival time and provide a new idea for the immunotherapeutic approach of gastric cancer (GC) (27). Compared with a low density of T cells, a higher density of T cells in the TME can better predict the prognosis of gastric cancer (28, 29). Salem et al. demonstrated that GARP restrains antitumor immunity by adjusting the function of Tregs in colorectal cancer (30). our analysis illustrated that the significant connection between GARP and TME was still worth exploring.

T helper cells, cytotoxic T cells, and Tregs are associated with T cell-mediated immune responses in the TME (31). Bioinformatic analysis illustrated that GARP expression was relevant to the immune groups of gastric cancer. In the colon cancer model, the loss of GARP in Treg leads to spontaneous inflammation and enteritis with high activation of CD4+ and CD8+ T cell, which has an important impact on immune surveillance (30). Our research illustrated GARP expression correlated with FOXP3+ Treg and CD4+ T cell infiltration and CTLA-4, and PD-L1 expression, whereas GARP expression had no remarkable connection with CD8+ T cell infiltration. Lucas et al. reported that the enhancement of this combination therapy did not depend on increasing the number of CD8+ T cells (7). Our study undoubtedly provided evidence for this result and also raised a question, specifically whether the antineoplastic effect of GARP affects the infiltration of CD4 + T cells. Despite the high expression of T cell markers indicate the improvement of progress in many cancers, no statistically significant correlation was observed in gastric cancer (24). A recent report has shown that T cells expressing immune checkpoints such as LAG3, PD1 may represent exhausted T-cell (32). In our analysis, the levels of CD4+ T cell and FOXP3+ Treg infiltration were significantly higher in patients with T3 gastric cancer than patients with T1 and T2 gastric cancer. We further found the expression of GARP in CD4+ T cells and analyzed that GARP+CD4+ T cells play a significant role in the prognosis of gastric cancer. The nomogram also showed that GARP+CD4+ T cell can predict the survival rate of GC patients together with other clinicopathological parameters. CD4 positive cells are likely to annotate as Tregs, dendritic cells, macrophages, or NK cells (27, 33). GARP may identify a special subgroup of T cells related to inferior prognosis. The variations in the phenotype of tumor-infiltrating immune cells and their relationship with prognosis highlight the clinical significance of the crosstalk between tumor cells and TME (34). However, more efforts are needed to determine whether T cell dysfunction is associated with GARP expression and poor prognosis of GC patients.

By analyzing the STRING database and our study, we also found an interaction between GARP, PD-L1 and, CTLA-4 expression. Researches have also found a significant correlation between PD-1+PD-L1+ T cells and Tregs. In animal experiments, combined therapy targeting GARP and PD-1 has achieved positive results (7, 35). A recent report suggests that combining checkpoint inhibitors with chimeric antigen receptor T-cells may also be of great significance in the treatment of cancer (27). GARP upregulation was related to the TGF-β signaling pathway. Evidence of TGF-β signaling in T cells has been found in melanoma specimens infiltrated by GARP-expressing T cells, indicating that inhibiting the activity of Treg-derived TGF-β1 using anti-GARP: TGF-β1 mAbs may effectively enhance CD8+ T cell-mediated antitumor immunity (7). Our study illustrated that the TGF-β signaling pathway was differentially enriched in the GARP high expression phenotype. Whether GARP affects the immune microenvironment of gastric cancer through the TGF-β signaling pathway is worthy of further exploration.

This study had several limitations. For example, the number of samples was limited, thus limiting the strength of our conclusions. In addition, we have not yet identified the regulatory pathway connecting GARP expression and CD4+ T cell, nor have we confirmed whether there are interactions among GARP, CTLA-4, and PD-L1. The combination of anti-GARP: TGF-β mAbs and PD-1 inhibitors can significantly enhance the effector ability of tumor T cells (36–38). Although remarkable progress has been made in cancer treatment by blocking CTLA-4 or PD-1 pathway with mAbs, most patients do not respond to therapy because of T cell-mediated primitive or acquired immune resistance to anti-tumor drugs (8, 39, 40). Combining checkpoint inhibitors with chimeric antigen receptor T-cell may also be of great significance in the treatment of cancer.

Taken together, our study proved that GARP is an independent influencing factor that is significantly upregulated in gastric cancer. We underlined the relationship between GARP and tumor-infiltrating T-cell. The phenotype of tumor-infiltrating immune cells was clinically meaningful, thereby providing a new idea for the immunotherapeutic approach of gastric cancer (30, 41).

## Data Availability Statement 

The datasets presented in this study can be found in online repositories. The names of the repository/repositories and accession number(s) can be found in the article/**Supplementary Material**.

## Author Contributions 

Conceptualization, JH. Data acquisition, SJ, YZ, XZ, BL, and PS. Formal analysis, SJ and YZ. Funding acquisition, JH. Methodology, XD and QW. Supervision, XZ. Validation, BL and PS. Visualization, SJ. Writing – original draft, SJ and YZ. Writing – review & editing, SJ. All authors contributed to the article and approved the submitted version.

## Funding

This work was supported by the National Natural Science Foundation of China (81874067), Jiangsu Provincial Department of Science and Technology (EB2018673), and the Scientific Research Project of Jiangsu Provincial Health Commission (H2017052).

## Conflict of Interest

The authors declare that the research was conducted in the absence of any commercial or financial relationships that could be construed as a potential conflict of interest.

## Publisher’s Note

All claims expressed in this article are solely those of the authors and do not necessarily represent those of their affiliated organizations, or those of the publisher, the editors and the reviewers. Any product that may be evaluated in this article, or claim that may be made by its manufacturer, is not guaranteed or endorsed by the publisher.

## References

[1] Van Cutsem E Sagaert X Topal B Haustermans K Prenen H. Gastric Cancer. Lancet (2016) 388(10060):2654–64. 10.1016/S0140-6736(16)30354-3 27156933

[2] Shah MA Ajani JA. Gastric Cancer–An Enigmatic and Heterogeneous Disease. JAMA (2010) 303(17):1753–4. 10.1001/jama.2010.553 20442394

[3] Kong R Zhang EB Yin DD You LH Xu TP Chen WM. Long Noncoding RNA PVT1 Indicates a Poor Prognosis of Gastric Cancer and Promotes Cell Proliferation Through Epigenetically Regulating P15 and P16. Mol Cancer (2015) 14:82. 10.1186/s12943-015-0355-8 25890171 PMC4399399

[4] Jin Z Jiang W Wang L. Biomarkers for Gastric Cancer: Progression in Early Diagnosis and Prognosis (Review). Oncol Lett (2015) 9(4):1502–8. 10.3892/ol.2015.2959 PMC4356326 25788990

[5] Fridman WH Pagès F Sautès-Fridman C Galon J. The Immune Contexture in Human Tumours: Impact on Clinical Outcome. Nat Rev Cancer (2012) 12(4):298–306. 10.1038/nrc3245 22419253

[6] Ma Y Pitt JM Li Q Yang H. The Renaissance of Anti-Neoplastic Immunity From Tumor Cell Demise. Immunol Rev (2017) 280(1):194–206. 10.1111/imr.12586 29027231

[7] de Streel G Bertrand C Chalon N Liénart S Bricard O Lecomte S. Selective Inhibition of TGF-β1 Produced by GARP-Expressing Tregs Overcomes Resistance to PD-1/PD-L1 Blockade in Cancer. Nat Commun (2020) 11(1):4545. 10.1038/s41467-020-17811-3 32917858 PMC7486376

[8] Ribas A Wolchok JD. Cancer Immunotherapy Using Checkpoint Blockade. Science (2018) 359(6382):1350–5. 10.1126/science.aar4060 PMC7391259 29567705

[9] Zhang Y Zhang Z. The History and Advances in Cancer Immunotherapy: Understanding the Characteristics of Tumor-Infiltrating Immune Cells and Their Therapeutic Implications. Cell Mol Immunol (2020) 17(8):807–21. 10.1038/s41423-020-0488-6 PMC7395159 32612154

[10] Yang H Yamazaki T Pietrocola F Zhou H Zitvogel L Ma Y. Improvement of Immunogenic Chemotherapy by STAT3 Inhibition. Oncoimmunology (2016) 5(2):e1078061. 10.1080/2162402X.2015.1078061 27057456 PMC4801443

[11] Batlle E Massagué J. Transforming Growth Factor-β Signaling in Immunity and Cancer. Immunity (2019) 50(4):924–40. 10.1016/j.immuni.2019.03.024 PMC7507121 30995507

[12] Stockis J Dedobbeleer O Lucas S. Role of GARP in the Activation of Latent TGF-β1. Mol Biosyst (2017) 13(10):1925–35. 10.1039/C7MB00251C 28795730

[13] Edwards JP Thornton AM Shevach EM. Release of Active TGF-β1 From the Latent TGF-β1/GARP Complex on T Regulatory Cells Is Mediated by Integrin β8. J Immunol (2014) 193(6):2843–9. 10.4049/jimmunol.1401102 PMC4157079 25127859

[14] Stockis J Liénart S Colau D Collignon A Nishimura SL Sheppard D. Blocking Immunosuppression by Human Tregs In Vivo With Antibodies Targeting Integrin αvβ8. Proc Natl Acad Sci USA (2017) 114(47):E10161–8. 10.1073/pnas.1710680114 29109269 PMC5703296

[15] Metelli A Salem M Wallace CH Wu BX Li A Li X. Immunoregulatory Functions and the Therapeutic Implications of GARP-TGF-Beta in Inflammation and Cancer. J Hematol Oncol (2018) 11(1):24. 10.1186/s13045-018-0570-z 29458436 PMC5819195

[16] Wang R Kozhaya L Mercer F Khaitan A Fujii H Unutmaz D. Expression of GARP Selectively Identifies Activated Human FOXP3+ Regulatory T Cells. Proc Natl Acad Sci USA (2009) 106(32):13439–44. 10.1073/pnas.0901965106 PMC2726405 19666573

[17] Wallace CH Wu BX Salem M Ansa-Addo EA Metelli A Sun S. B Lymphocytes Confer Immune Tolerance via Cell Surface GARP-TGF-β Complex. JCI Insight (2018) 3(7):e99863. 10.1172/jci.insight.99863 PMC5928869 29618665

[18] Metelli A Wu BX Fugle CW Rachidi S Sun S Zhang Y. Surface Expression of Tgfβ Docking Receptor GARP Promotes Oncogenesis and Immune Tolerance in Breast Cancer. Cancer Res (2016) 76(24):7106–17. 10.1158/0008-5472.CAN-16-1456 PMC5504525 27913437

[19] Ru B Wong CN Tong Y Zhong JY Zhong SSW Wu WC. TISIDB: An Integrated Repository Portal for Tumor-Immune System Interactions. Bioinformatics (2019) 35(20):4200–2. 10.1093/bioinformatics/btz210 30903160

[20] Chen B Khodadoust MS Liu CL Newman AM Alizadeh AA. Profiling Tumor Infiltrating Immune Cells With CIBERSORT. Methods Mol Biol (2018) 1711:243–59. 10.1007/978-1-4939-7493-1_12 PMC5895181 29344893

[21] Subramanian A Tamayo P Mootha VK Mukherjee S Ebert BL Gillette MA. Gene Set Enrichment Analysis: A Knowledge-Based Approach for Interpreting Genome-Wide Expression Profiles. Proc Natl Acad Sci USA (2005) 102(43):15545–50. 10.1073/pnas.0506580102 PMC1239896 16199517

[22] von Mering C Huynen M Jaeggi D Schmidt S Bork P Snel B. STRING: A Database of Predicted Functional Associations Between Proteins. Nucleic Acids Res (2003) 31(1):258–61. 10.1093/nar/gkg034 PMC165481 12519996

[23] Zhang X Shi M Chen T Zhang B. Characterization of the Immune Cell Infiltration Landscape in Head and Neck Squamous Cell Carcinoma to Aid Immunotherapy. Mol Ther Nucleic Acids (2020) 22:298–309. 10.1016/j.omtn.2020.08.030 33230435 PMC7522342

[24] Fakih M Ouyang M Wang C Tu C Gozo TY Cho MC. Immune Overdrive Signature in Colorectal Tumor Subset Predicts Poor Clinical Outcome. J Clin Invest (2019) 129(10):4464–76. 10.1172/JCI127046 PMC6763253 31524634

[25] Bertolini TB Biswas M Terhorst C Daniell H Herzog RW Piñeros AR. Role of Orally Induced Regulatory T Cells in Immunotherapy and Tolerance. Cell Immunol (2021) 359:104251. 10.1016/j.cellimm.2020.104251 33248367 PMC8919860

[26] Darvin P Toor SM Sasidharan Nair V Elkord E. Immune Checkpoint Inhibitors: Recent Progress and Potential Biomarkers. Exp Mol Med (2018) 50(12):1–11. 10.1038/s12276-018-0191-1 PMC6292890 30546008

[27] Autio M Leivonen SK Bruck O Mustjoki S Jorgensen JM Karjalainen-Lindsberg ML. Immune Cell Constitution in the Tumor Microenvironment Predicts the Outcome in Diffuse Large B-Cell Lymphoma. Haematologica (2020) 106(3):718–29. 10.3324/haematol.2019.243626 PMC7927991 32079690

[28] Cai WY Dong ZN Fu XT Lin LY Wang L Ye GD. Identification of a Tumor Microenvironment-Relevant Gene Set-Based Prognostic Signature and Related Therapy Targets in Gastric Cancer. Theranostics (2020) 10(19):8633–47. 10.7150/thno.47938 PMC7392024 32754268

[29] Kim JW Nam KH Ahn SH Park DJ Kim HH Kim SH. Prognostic Implications of Immunosuppressive Protein Expression in Tumors as Well as Immune Cell Infiltration Within the Tumor Microenvironment in Gastric Cancer. Gastric Cancer (2016) 19(1):42–52. 10.1007/s10120-014-0440-5 25424150

[30] Salem M Wallace C Velegraki M Li A Ansa-Addo E Metelli A. GARP Dampens Cancer Immunity by Sustaining Function and Accumulation of Regulatory T Cells in the Colon. Cancer Res (2019) 79(6):1178–90. 10.1158/0008-5472.CAN-18-2623 PMC6420855 30674536

[31] Ostroumov D Fekete-Drimusz N Saborowski M Kühnel F Woller N. CD4 and CD8 T Lymphocyte Interplay in Controlling Tumor Growth. Cell Mol Life Sci (2018) 75(4):689–713. 10.1007/s00018-017-2686-7 29032503 PMC5769828

[32] Xiong H Mittman S Rodriguez R Pacheco-Sanchez P Moskalenko M Yang Y. Coexpression of Inhibitory Receptors Enriches for Activated and Functional CD8(+) T Cells in Murine Syngeneic Tumor Models. Cancer Immunol Res (2019) 7(6):963–76. 10.1158/2326-6066.CIR-18-0750 31064777

[33] O’Doherty U Steinman RM Peng M Cameron PU Gezelter S Kopeloff I. Dendritic Cells Freshly Isolated From Human Blood Express CD4 and Mature Into Typical Immunostimulatory Dendritic Cells After Culture in Monocyte-Conditioned Medium. J Exp Med (1993) 178(3):1067–76. 10.1084/jem.178.3.1067 PMC2191184 8102389

[34] Zeng D Li M Zhou R Zhang J Sun H Shi M. Tumor Microenvironment Characterization in Gastric Cancer Identifies Prognostic and Immunotherapeutically Relevant Gene Signatures. Cancer Immunol Res (2019) 7(5):737–50. 10.1158/2326-6066.CIR-18-0436 30842092

[35] Cuende J Liénart S Dedobbeleer O van der Woning B De Boeck G Stockis J. Monoclonal Antibodies Against GARP/TGF-β1 Complexes Inhibit the Immunosuppressive Activity of Human Regulatory T Cells In Vivo. Sci Transl Med (2015) 7(284):284ra56. 10.1126/scitranslmed.aaa1983 25904740

[36] Hui E Cheung J Zhu J Su X Taylor MJ Wallweber HA. T Cell Costimulatory Receptor CD28 is a Primary Target for PD-1-Mediated Inhibition. Science (2017) 355(6332):1428–33. 10.1126/science.aaf1292 PMC6286077 28280247

[37] Arasanz H Gato-Cañas M Zuazo M Ibañez-Vea M Breckpot K Kochan G. PD1 Signal Transduction Pathways in T Cells. Oncotarget (2017) 8(31):51936–45. 10.18632/oncotarget.17232 PMC5584302 28881701

[38] Dahan R Sega E Engelhardt J Selby M Korman AJ Ravetch JV. Fcγrs Modulate the Anti-Tumor Activity of Antibodies Targeting the PD-1/PD-L1 Axis. Cancer Cell (2015) 28(3):285–95. 10.1016/j.ccell.2015.08.004 26373277

[39] Zaretsky JM Garcia-Diaz A Shin DS Escuin-Ordinas H Hugo W Hu-Lieskovan S. Mutations Associated With Acquired Resistance to PD-1 Blockade in Melanoma. N Engl J Med (2016) 375(9):819–29. 10.1056/NEJMoa1604958 PMC5007206 27433843

[40] Yang H Xia L Chen J Zhang S Martin V Li Q. Stress-Glucocorticoid-TSC22D3 Axis Compromises Therapy-Induced Antitumor Immunity. Nat Med (2019) 25(9):1428–41. 10.1038/s41591-019-0566-4 31501614

[41] Rachidi S Metelli A Riesenberg B Wu BX Nelson MH Wallace C. Platelets Subvert T Cell Immunity Against Cancer *via* GARP-Tgfβ Axis. Sci Immunol (2017) 2(11):eaai7911. 10.1126/sciimmunol.aai7911 28763790 PMC5539882

