# Supplementary material for: GARP Correlates With Tumor-Infiltrating T-Cells and Predicts the Outcome of Gastric Cancer
Source: Front Immunol. 2021 Aug 6;12:660397. doi: 10.3389/fimmu.2021.660397 (PMC8378229; doi:10.3389/fimmu.2021.660397)
Supplement: Supplementary file 1 [file Table_1.docx]

Supplementary Material

# Supplementary Figures and Tables

## Supplementary Figure 1


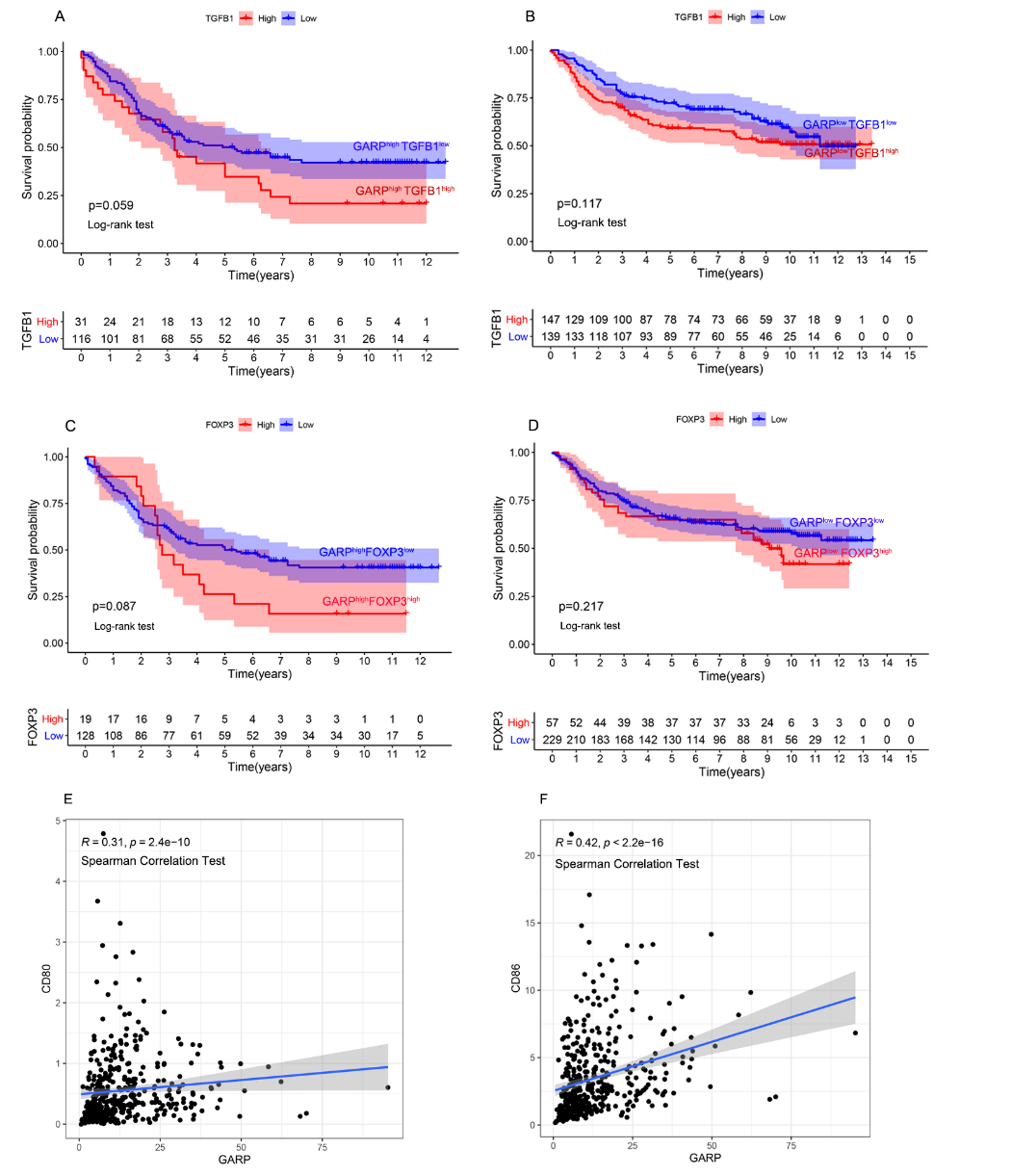


**Supplementary Figure 1** (**A)** Kaplan–Meier curves for TGF-ꞵ1 low and high group in GARP high group. The cutoff point was based on the median. (**B)** Kaplan–Meier curves for TGF-ꞵ1 low and high group in GARP low group. **C** Kaplan–Meier curves for FOXP3 low and high group in GARP high group. (**D)** Kaplan–Meier curves for FOXP3 low and high group in GARP low group. (**E-F)** The relationships between GARP and CD80 expression, GARP and CD86 expression in TCGA cohort.

## Supplementary Figure 2


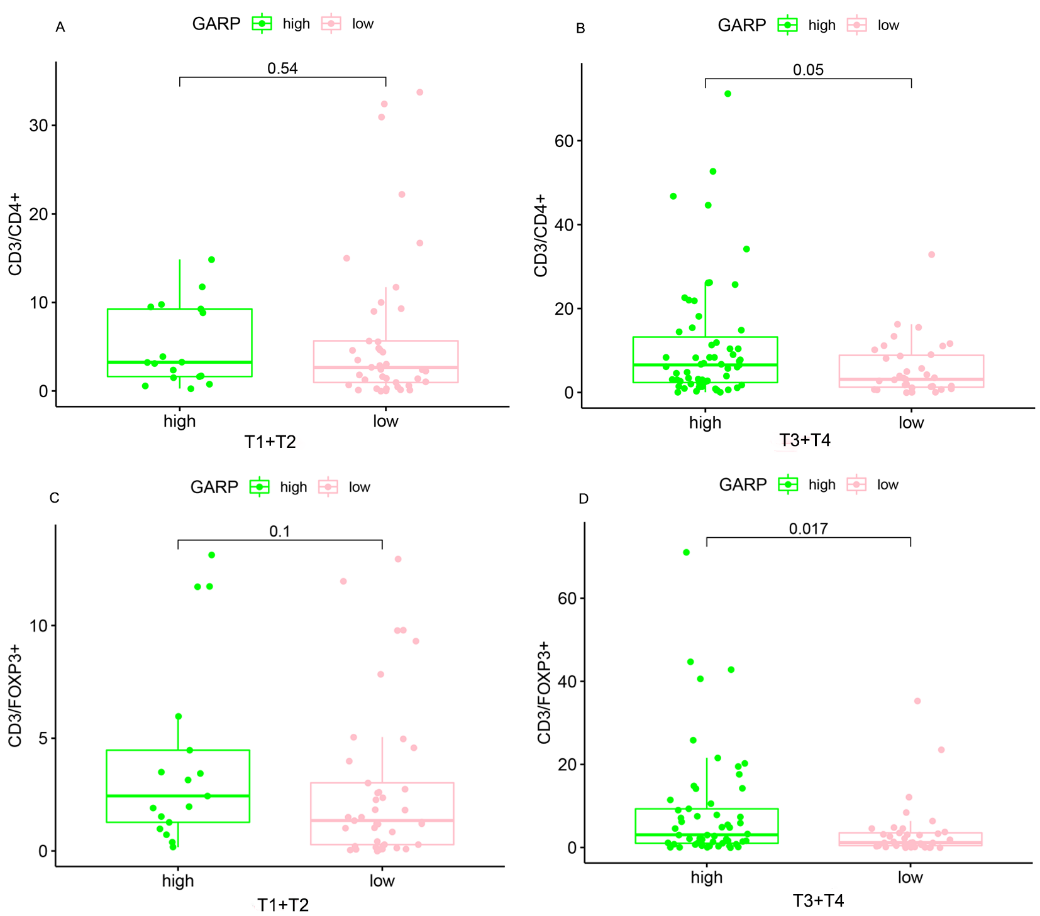


**Supplementary Figure 2 (A-B)** The infiltration of CD4+ T cells in the GARP high/low group. **(A)** Tumor size (T1+T2). **(B)** Tumor size (T3+T4). (**C-D)** The infiltration of Tregs in the GARP high/low group. **(C)** Tumor size (T1+T2). **(D)** Tumor size (T3+T4).

## Supplementary Table

Bonferroni adjustment for multiple comparisons of clinicopathologic characteristics in the GARP-high group and the GARP-low group.

| T | P - Value | α = 0.0125 |
| --- | --- | --- |
| Tis+T1 vs T2 | 0,026 |  |
| T1 vs T3 | < 0.001 |  |
| T1 vs T4 | 0.001 |  |
| T2 vs T3 | 0.263 |  |
| T2 vs T4 | 0.053 |  |
| T3 vs T4 | 0.196 |  |
| N |  | α = 0.0125 |
| N0 vs N1 | 0.645 |  |
| N0 vs N2 | 0.047 |  |
| N0 vs N3 | 0.224 |  |
| N1 vs N2 | 0.301 |  |
| N1 vs N3 | 0.610 |  |
| N2 vs N3 | 0.636 |  |
| TNM |  | α = 0.0125 |
| Ⅰ vs Ⅱ | 0.026 |  |
| Ⅰ vs Ⅲ | < 0.001 |  |
| Ⅰ vs Ⅳ | 0.001 |  |
| Ⅱ vs Ⅲ | 0.263 |  |
| Ⅱ vs Ⅳ | 0.053 |  |
| Ⅲ vs Ⅳ | 0.196 |  |
| Differentiation |  | α = 0.0167 |
| Poor vs middle | 1.000 |  |
| Poor vs well | 0.728 |  |
| Middle vs well | 0.286 |  |

α, inspection level; T, tumor size; N, lymph node metastasis; M, distant metastasis.
